# PROTOCOL: Counter‐narratives for the prevention of violent radicalisation: A systematic review of targeted interventions

**DOI:** 10.1002/CL2.202

**Published:** 2018-09-24

**Authors:** Sarah L. Carthy, Colm B. Doody, Denis O'Hora, Kiran M. Sarma

## Background

### The problem, condition or issue

Since the start of the 21^st^ century, academics and counter‐terrorism practitioners have increasingly sought to better understand the process(es) through which individuals transition from non‐violence into terrorism (Dawson, 2017; Europol, 2016; Horgan, 2008; European Parliament, 2017). This progression, believed to be characterised by the movement from an unfocused state of uncertainty towards a narrower, unambiguous state of clarity (Horgan, 2008), is often referred to as ‘violent radicalisation’. At least in part, research into violent radicalisation has been motivated by a desire to identify ‘phase specific intervention strategies’ in the radicalisation process (Horgan, Gill, Bouhana, James Silver, & Corner, 2016).; Identification of these “pinch‐points” may provide opportunity for intervention, helping redirect individuals onto a trajectory towards non‐violence (p. 53).

One core focus of enquiry by social scientists has been the role of narratives in the process of violent radicalisation (e.g. terrorist organisations’ use of narratives for propagandistic purposes (Braddock, 2015)). Narratives here refer to recollections of events which happen in sequence (Barthes &Duisit, 1975; Genette, 1982) with characters that can cause changes (Richardson, 2002), neatly contained within an identifiable beginning, middle and end (Hinyard&Kreuter, 2007, p. 778). The following is a popular religious narrative found in numerous religious texts (including the Qur'an and The Torah);

Moses, having pleaded with The Pharaoh of Egypt to release the Hebrews and accept the One True God, threatened Pharaoh with divine retribution. The Pharaoh was arrogant and ignored Moses’ warning. As promised God punished Pharaoh with several disasters such as drought, famine, disease, locusts, lice and frogs brought upon his own people.

The objectives of such narratives are to present a social construction of the world which serves the interest of those legitimizing violence. In this instance, one can justify the murder of innocent civilians as retribution for not accepting a particular worldview.

### The intervention

To tackle these violent messages, researchers have considered the potential for counter‐narratives messaging. Briggs and Reve (2013) differentiate between three approaches to counter‐messaging, including alternative narratives, government strategic communications and counter‐narratives. Whilst the former attempt to promote positive stories about social values such as openness and tolerance (alternative narratives) or raise awareness about positive government initiatives (government strategic communications), counter‐narratives are conceptually different. By deconstructing, discrediting and demystify violent extremist messaging, counter‐narratives present people with alternative social constructions to those presented by the propagandist. They are described as tailored responses which challenge the themes intrinsic to extremist narratives (Braddock & Horgan, 2016, p. 386). Taking the narrative above, for instance, a counter‐narrative could state that;

In the divine retribution, it was God, not Moses, who rained terror on his people. In this way, the murder of innocent civilians by man is nothing more than the very behaviour for which Pharaoh, a man, was punished. Only God can offer divine retribution.

Through challenging such narratives, counter‐narratives may help individuals make more informed decisions through deeper consideration of, for example, the validity of certain arguments, the rationality of hatred or the legitimacy of violent action (Goodall Jr, 2010).

In applied research, counter‐narratives have attempted to challenge prevailing narratives in a number of areas, including discrediting inaccurate historical narratives (Luckhurst, 2012) and news coverage (Lee, 2012). In the field of violent extremism, the concept of a counter‐narrative is not new. Following the 9/11 attacks, counter‐narratives were used to target attitudes about the treatment of Muslims in the United States as part of the US State Department's ‘Shared Values’ initiative (Fullerton & Kendrick, 2006). The campaign included five mini‐documentaries which were broadcast in Muslim majority countries, showing the happy lives of American Muslims, living freely in America without persecution.

More recently, the Organization for Security and Co‐operation in Europe (OSCE) incorporates counter‐narratives to promote interfaith dialogue and for young people at risk of violent radicalisation. The campaign also uses documentary style videos promoting themes of acceptance and tolerance (targeting polarised attitudes) under the #UnitedCVE hashtag.

Finally, a more direct example of the use of counter‐narratives to discredit terrorist propaganda is the Anti‐ISIL Global Coalition (funded by the European Union's Strategic Communication Task Force). These initiatives use counter‐narratives to degrade, and ultimately defeat, Daesh by targeting romanticised attitudes towards the group with online articles such as ‘The Truth About Life Under Daesh in Raqqa’ (The Global Coalition Against Daesh, January 11, 2018).

### How the intervention might work

Although the evidence‐base for the development of counter‐narratives is limited, there have been several contributions to our general understanding of attitude and behaviour change in respect to violent radicalisation and terrorism. These contributions have come from psychology (Ginges, 2009; Taylor & Horgan, 2006), psychiatry (Bhui, Everitt, & Jones, 2014), sociology (Bouhana&Wikstrom, 2011) and political science (Sageman, 2004).

Psychological research would suggest that narratives can influence both attitudes and behaviour in a number of ways. Theories informed by aspects of communication such as the credibility of the source, message repetition, individual differences in the “receiver” or “target” (Anderson &Hovland, 1957; Hovland, Janis, & Kelley, 1953), the presence of dissonance or ‘mental discomfort’ (Aronson, Turner, &&Carlsmith, 1963; Bochner&Insko, 1966, p. 614; Festinger, 1957) as well as the target's initial, attitudinal position (Sherif&Sherif, 1967; Sherif, Sherif, &Nebergall, 1965) have contributed to our understanding of the persuasive power of narratives in several contexts. However, our understanding of persuasive communication can also inform our attempts at countering it.

In line with discrepancy models, Brock (1967) found that discrepancy causes reduced belief change through the creation of counter‐arguments. Discrepancy, Brock argues, causes more elaborate processing through a central (rather than peripheral) route which naturally leads to more counter‐arguments and inevitably reduced attitude change. In other words, by encouraging more elaborate processing, the likelihood of considering several sides of a narrative is increased and the rational (Halverson, Corman, & Goodall Jr, 2011), simplistic (Kesterson in Cabayan, V., &Yandura, 2013) and singular narrative approach to persuasion (which has enjoyed immense success in violent radicalisation) is rendered less effective.

Alternatively, the construction of a counter‐narrative could be more heavily informed Berlo's aspects of communication whereby differentiations are made between persuasive communication as response shaping, response reinforcing or response changing. A response reinforcing process, whereby the message seeks to reinforce currently held beliefs and make them more resistant to change (Miller &Burgoon, 1973) rather than a response‐changing process (whereby the message seeks to actively challenge an existing message) is more likely to be effective (Bettinghaus, 1973; Brembeck& Howell, 1952; Cronkhite, 1969; Dillard & Shen, 2013; Scheidel, 1967 as cited in Dillard and Shen).

### Why it is important to do the review

In conjunction with the Partnership for Conflict, Crime & Security Research, Ferguson (2016) published a “horizon scan” (p. 5) of the research landscape in relation to the research question “how can media and communications be used to counter identity‐based violence (IBV) or Violent Extremism (VE)?” (p. 2); the report details the state of research in counter‐narratives and, although it provides insight into the area, it does not synthesise the experimental evidence on the effects of counter‐narrative interventions but, rather, cites government‐led and grassroots initiatives akin to those mentioned earlier in this paper. These initiatives, as mentioned, starkly lack an evaluative component, hence, Ferguson's conclusion that there is insufficient evidence to demonstrate the efficacy and effectiveness of counter‐narrative strategies for countering violent extremism (CVE). The current authors, as mentioned, do not believe this is the case, thus, prompting the current review.

Briggs and Reves (2013) authored a similar report which offered an overview of different “counter‐messaging” initiatives, including counter‐narratives. Rather than assessing their effectiveness, Briggs and Reve drew on theory in persuasive communication to highlight the potential of these attempts. For example, message credibility was discussed in relation to message dissemination by former violent extremists, government departments or survivors of violent extremism (pp. 17‐18). Again, many of the cited attempts lacked an evaluative component so it is unsurprising that this theoretical approach was used.

In the second half of Ferguson's report, she discusses alternative approaches for which there is a stronger evidence base such as inoculation (the cited study attempted to make participants ‘immune’ to hate speech, for example) and alternative narratives (offering participants an alternative world view, but not directly referencing an established narrative). In relation to these alternative approaches, Ferguson assesses that this literature base is also limited and, within these studies, the measured outcomes do not directly address violence prevention (p. 17), thus excluding many from the current review. Furthermore, by the definition used in this review, many of the cited alternative approaches are not counter‐narratives.

For this reason, although these papers will be included in Stage 3 of the search strategy, the scope and rigour of the current review will attempt to expand upon these “horizon scan” syntheses of counter‐narrative studies. Acknowledging that it is important to discuss all grassroots, government‐led, empirical and non‐empirical counter‐narrative attempts, this review aims to go further by providing a synthesis of the effectiveness of targeted counter‐narrative interventions. In this way, the quality of the evidence, rather than the types of attempts, can be quantified.

In terms of evidence, syntheses have been made of the effectiveness of similar approaches in other fields. For example, (Stice& Shaw, 2004) provided meta‐analytic evidence on the use of a proximal approach called dissonance‐based interventions (DBI) which encourages individuals to adopt a way of thinking which contradicts their current way of thinking (e.g. challenging social constructions of ‘beauty’ or ‘thinness’). Furthermore, Chan, Jones, Hall Jamieson, and Albarracin (2017) provided meta‐analytic evidence on the factors underlying effective counter‐arguing or ‘debunking’ of misinformation (for example, conspiracy theories or ‘fake news’). However, to date, there has been no synthesis of the effectiveness of counter‐narrative interventions for the prevention of violent radicalisation. This review seeks to address this.

The review will contribute to existing theory and evidence on counter‐narrative interventions and allow researchers and practitioners to better understand message style and content, psychological fulcra for change targeted and, importantly, the effectiveness of this approach in reducing outcomes related to violent radicalisation. In doing so, the review may aid those tasked with the design of counter‐narratives and, ultimately, help those at risk of violent radicalisation to more critically consider the validity of the messages being communicated through extremist narratives.

## Objectives

The objective of this review is to provide a synthesis of the effectiveness of counter‐narratives in reducing the risk of violent radicalisation into terrorism. The review question that will guide this research is:

What is the impact of counter‐narratives on violent radicalisation (primary outcomes) and/or risk factors for violent radicalisation (secondary outcomes)?

## Methodology

Note: when completing this section, please refer to the Campbell Collaboration Systematic Reviews: Policies and Guidelines. At a minimum, this section should include the information under each of the sub‐sections below:

### Criteria for including and excluding studies

#### Types of study designs

Studies adopting an experimental design will be included in the review. In other words, studies in which causality can be independently determined through experimental manipulation using standardised procedures and random assignment. This includes studies where at least one of the independent variables involves comparing a counter‐narrative to a control or comparison exposure (e.g. two‐group between‐subjects design) before measuring outcomes. Other forms of experimental designs that we anticipate may be present in the literature include factorial designs, with more than one independent variable (e.g. pre‐post as a within‐subjects variable, and exposure (e.g. present/absent) as a between‐subjects variable). Quasi‐experimental designs (e.g. interrupted time‐series designs) and other forms of designs in which causality cannot be independently determined will be excluded from the review. However, quasi‐experimental studies which include base‐line measures and a comparison group will be included. With all included studies, risk of bias and quality will determine interpretation as part of the meta‐analysis or, alternatively, as part of the narrative synthesis.

#### Types of interventions

To be included in the review, studies must; 1) investigate the results of the implementation of a counter‐narrative to challenge an existing narrative and; 2) this existing narrative must promote violent extremism.

The existing narrative may be already present in the sample before experimental manipulation of the counter‐narrative. In the United States, for example, two studies were conducted by Allhabash and Wise (2012, 2015) in an American University sample, exposing participants to a narrative which challenged the pro‐Israeli perspective in the Israeli‐Palestinian conflict, classified as a risk factor for ethnic extremism. In both studies, the established narrative of participants was confirmed through pre‐exposure attitude measures before randomly exposing half the participants to the experimental condition and half to the control condition.

In some cases, the established narrative may be experimentally introduced in an attempt to more stringently control the manipulation. Variations of studies which expose a sample to narrative before exposing them to a counter‐narrative and subsequently measuring outcomes across experimental (counter‐narrative) and control (no counter‐narrative) would constitute a counter‐narrative intervention.

In other words, as the review is interested in changes following exposure to a counter‐narrative (rather than a narrative), studies which expose participants to a narrative which does not challenge a pre‐existing or experimentally introduced narrative will be excluded. For example, (Davenport, 2013) exposed 224 introductory psychology students to a news clip about a terrorist attack in which mortality salience was manipulated before stereotyping attitudes were measured. However, the exposure material was not designed to challenge an established narrative, nor was an established narrative ever gauged or experimentally introduced. For this reason, the study can be characterised as measuring the effects of exposure to a narrative. It does not, however, measure the effects of exposure to a counter‐narrative.

It is important to highlight that interventions which expose participants to a counter‐narrative after exposure to a narrative (therapeutic interventions) and interventions which expose participants to a counter‐narrative before exposure to a narrative (preventative intervention) will both be included in the review.

Furthermore, exposure to the counter‐narrative(s) must be intended to reduce propensity towards violent radicalisation, rather than an unrelated, extraneous outcome (e.g. blood pressure).

#### Types of outcome measures

Studies investigating the connection between exposure to a counter‐narrative and propensity towards violent radicalisation will be included. These may include primary outcomes (e.g. engagement in violent extremism or providing support to violent extremist groups) and secondary outcomes (e.g. adversarial stereotypes, outgroup feelings, attitudes towards violence, etc.).

### Search strategy

Potentially relevant literature will be identified through a four‐stage search strategy, which will comprise:

Stage 1) Targeted keyword searches on a list of relevant databases (Table 2).
Stage 2) Hand searches of several research and professional agencies’ outputs and publications (Table 3).
Stage 3) A review of reference lists of conceptual papers and books on the topic of counter‐narratives in counter‐terrorism (Table 4).
Stage 4) Contacting experts in the area (Table 5).

In conjunction with a specialist librarian at the National University of Ireland Galway, a comprehensive list of search terms has been developed (see Table 1). The search strategy will be replicated by a co‐author upon selection of the studies (controlling for date changes). If there is more than 10% discrepancy between selections, both authors will apply the search strategy together and resolve any differences.

### Description of screening process

All identified literature will undergo a three‐stage screening process. The titles of all literature will be screened, and papers excluded based, on the exclusion criteria. Studies will be excluded if they do not:

1. Deliver an intervention which challenges an existing or experimentally introduced narrative with a counter‐narrative to reduce propensity towards violent extremism.
2. Measure outcomes related to violent extremism.
3. Adopt an experimental design (i.e. experimental manipulation using standardised procedures and random assignment) or quasi‐experimental designs (which include base‐line measures and a comparison group).

During this stage, papers will be rejected if, based on the title, they are clearly not eligible. The abstracts of the remaining literature will then be screened, again with studies excluded according to the exclusion criteria. Finally, the full texts of remaining studies will be screened according to the inclusion criteria, producing the final set of studies to be included in the review.

The above will be performed by the primary researcher (SC). A second screener (CD) will repeat the three‐stage screening process. As mentioned, if there is more than 10 percent discrepancy between both screeners’ included studies, both screeners will repeat the third stage of the screening process with both screeners’ final studies (based on the inclusion criteria) before reaching a consensus. See Coding Lists a) and b) (Appendix 2) for title and abstract exclusion criteria and full title inclusion criteria.

### Criteria for determination of independent findings

In relation to effectiveness, it is expected that some studies will report impact estimates in relation to various outcomes related to violent radicalisation. In order to ensure that the impact for each intervention type and outcome are derived from statistically independent findings, the following will apply:

1. In the case of multiple comparable outcomes and effect sizes within the same study (e.g. a study examining the effects of a counter‐narrative on explicit attitudes and explicit stereotyping), the outcome which; a) uses the measure with the highest validity as well as; b) is most proximal to the desired outcome (primary or secondary risk factors for violent radicalisation) will be used.
2. In the case of more than one time‐period, the estimate with the lowest risk of bias will be used. If the risk of bias is equal, the most recent time‐period will be used.
3. In the case of a study reporting more than one effect size for an outcome by subgroup (for example, gender or ethnicity), groups will be combined to calculate a weighted average where possible. Appropriate adjustments will be made to variances and standard errors.

### Details of study coding categories

For data extraction, a coding tool has been developed to isolate; a) descriptive information (including intervention design) as well as; b) information that allow the effects of the intervention on the outcome variables of interest to be represented. A full coding scheme is provided as Appendix 3 with a singular example. A risk of bias analysis will be conducted according to the Cochrane Effective Practice and Organisation of Care Review Group (EPOC) Data Collection Checklist. A quality analysis will be conducted according to the GRADE assessment of study limitations.

The primary researcher (SC) will code all of the studies (including risk of bias and quality assessment), and a second coder will double code all studies for risk of bias and quality (CD). If there are more than 10 percent discrepancy in critical fields, both coders will assess the discrepant studies and ensure the differences are resolved.

### Statistical procedures and conventions/data synthesis

Although we are limiting our criteria to experimental research (alongside the specified quasi‐experimental designs), it may be the case that certain outcomes cannot be synthesised in a meta‐analysis, but must be synthesised narratively. In line with the Campbell MECCIR standards, if a quantitative synthesis is not planned, or if it is not possible, authors must plan the specific methods to narratively synthesize the results of the included studies. We intend to:

1. Present a detailed summary of the results of each study, discussing relevant features of the intervention and research design and presenting effect sizes and 95% confidence intervals;
2. Where possible, we will use meta‐analytic techniques to combine data from different studies. A random effects meta‐analysis will be conducted using standardized mean differences between conditions (e.g. counter‐narrative vs. control), reporting on comparable outcomes where possible. For example, “implicit attitudes” as a general outcome could be compared with both the implicit association task (IAT) and the affective misattribution procedure (AMP). Reported effect sizes (preferably medium to large) will be transformed into standardised mean differences for outcomes employing continuous measures. Errors and 95% confidence intervals will be reported in each case. The statistical program Review Manager 5 (RevMan 5) will be used.
3. For studies not included in the meta‐analysis, the results will be synthesised across two headings ‘Key outcomes across studies’ and ‘Assessing differences in outcomes across studies’.

### Assessment of heterogeneity

Heterogeneity will be tested using the I‐squared statistic. The tau‐squared statistic for random‐effects meta‐analysis will also be reported.

### Assessment of publication bias

Using sub‐group analysis of published and unpublished studies, we will examine the possible effects of publication bias. Although it is unlikely we will find 10 or more studies for statistical testing, funnel graphs will be used if possible (Egger, 1997).

## Review authors

**Lead review author**:

**Table:** 

Name:	Sarah L. Carthy
Title:	Miss
Affiliation:	National University of Ireland Galway
Address:	University Road
City, State, Province or County:	Galway
Post code:	
Country:	Ireland
Phone:	0862124351
Email:	Sarah.l.carthy@gmail.com
**Co‐author(s):**	
Name:	Colm B. Doody
Title:	National University of Ireland Galway
Affiliation:	University Road
Address:	Galway
City, State, Province or County:	
Post code:	Ireland
Country:	
Phone:	Colm.doody@gmail.com
Email:	Mr
Name:	Denis O'Hora
Title:	Dr.
Affiliation:	NUI Galway
Address:	National University of Ireland Galway
City, State, Province or County:	Galway
Post code:	
Country:	Ireland
Phone:	
Email:	Denis.ohora@nuigalway.ie
Name:	Kiran M. Sarma
Title:	Dr.
Affiliation:	National University of Ireland Galway
Address:	University Road
City, State, Province or County:	Galway
Post code:	
Country:	Ireland
Phone:	
Email:	Kiran.sarma@nuigalway.ie

## Roles and responsibilities

Sarah Carthy is a PhD student at the National University of Ireland Galway, under the supervision of Dr Kiran Sarma. Sarah Carthy will coordinate and conduct the review. Colm Doody will act as second coder for the study selection process. Dr O'Hora and Dr Sarma will supervise the review.

## Sources of support

Funding has been provided by the Irish Research Council (IRC) and Child and Youth Research programme, National University of Ireland Galway.

## Declarations of interest

None.

## Preliminary timeframe

Approximate date for submission of the systematic review: September 2018.

## Plans for updating the review

This review, once completed, will be updated every two years to include additional study data. The primary author will take the lead in updating this review.

## AUTHOR DECLARATION

### Authors’ responsibilities

By completing this form, you accept responsibility for preparing, maintaining and updating the review in accordance with Campbell Collaboration policy. Campbell will provide as much support as possible to assist with the preparation of the review.

A draft review must be submitted to the relevant Coordinating Group within two years of protocol publication. If drafts are not submitted before the agreed deadlines, or if we are unable to contact you for an extended period, the relevant Coordinating Group has the right to de‐register the title or transfer the title to alternative authors. The Coordinating Group also has the right to de‐register or transfer the title if it does not meet the standards of the Coordinating Group and/or Campbell.

You accept responsibility for maintaining the review in light of new evidence, comments and criticisms, and other developments, and updating the review at least once every five years, or, if requested, transferring responsibility for maintaining the review to others as agreed with the Coordinating Group.

### Publication in the Campbell Library

The support of the Coordinating Group in preparing your review is conditional upon your agreement to publish the protocol, finished review, and subsequent updates in the Campbell Library. Campbell places no restrictions on publication of the findings of a Campbell systematic review in a more abbreviated form as a journal article either before or after the publication of the monograph version in Campbell Systematic Reviews. Some journals, however, have restrictions that preclude publication of findings that have been, or will be, reported elsewhere and authors considering publication in such a journal should be aware of possible conflict with publication of the monograph version in Campbell Systematic Reviews. Publication in a journal after publication or in press status in Campbell Systematic Reviews should acknowledge the Campbell version and include a citation to it. Note that systematic reviews published in Campbell Systematic Reviews and co‐registered with Cochrane may have additional requirements or restrictions for co‐publication. Review authors accept responsibility for meeting any co‐publication requirements.

**I understand the commitment required to undertake a Campbell review, and agree to publish in the Campbell Library. Signed on behalf of the authors:**

**Form completed by:**

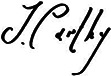

**Date: 9 August 2018**

## Appendix 1. Search strategy

**Table 1:** Search terms

**Search domain**	**Topical domain**	**Key Words**
Title or Abstract	Intervention *(‘counter‐narrative’)*	(Alter‐messaging OR Alternative framing OR Anti‐messaging OR anti‐radicalism message OR anti‐radicalization message OR Anti‐terrorist campaign OR Anti‐violence campaign OR Argument scrutiny OR attitude‐change OR Citizen messenger OR Common narrative OR Contesting narratives OR Countering ideological support for extremism OR Counter analogy OR Counter‐argument, Counter‐attitudinal OR Counter‐campaign OR Counter example OR Counter‐ideological OR Counter‐message OR Counter‐messaging campaign OR Counter‐messaging intervention OR Counter‐messaging interventions OR Counter‐messaging strategy OR Counter‐messenger OR Counter‐narrative OR Counter‐narrative campaign OR Counter‐narrative message OR counter‐radicalisation OR counter‐recruitment OR counter‐speech OR counter‐strategy OR countering campaign OR countering materials OR ideological counterpoint OR strategic narrative OR narrative intervention OR narrative transportation OR public diplomacy OR rhetorical education OR Security narrative OR strategic communication OR Ideological response)
Title or Abstract AND Full Text	Research area (*‘counter‐terrorism’*)	(Anti‐colonialism OR Anti‐imperialist OR Anti‐terror OR Anti‐terrorism OR Battle of Ideas OR Conflict OR Conflict resolution OR Counter‐terrorism OR counter‐radicalization OR Countering Violent Extremism OR CVE OR Deradicalisation OR Deradicalization OR De‐radicalisation OR De‐radicalization OR Disengagement OR Extremism OR Ideological distortion OR ideological distortions OR Ideological battle OR Ideological War OR Indoctrination OR intellectual activist OR Islamic terrorism OR Islamist terrorism OR militant activist OR Online radicalisation OR Recruit OR Radical OR Radical group OR Radical movement OR Radicalisation OR Radicalism OR Security OR Terrorism OR Terrorist action OR violence OR Violent Extremism OR War OR War of ideas OR Terrorist threat OR Terrorist incident OR Terrorist ideology OR Terrorist sympathiser OR Violent extremism online OR Violent extremist ideology OR Violent extremist message)
Full Text	Problem *(‘terrorist narrative’)*	(Alternative historical accounts OR Extremist propaganda OR Extremist sympathiser OR Audio‐visual production OR Branding OR Collective memory OR Digital communication OR Extremism online OR Extremist argument OR Extremist content online OR Extremist ideology OR Extremist message OR Extremist narrative OR Anti‐American OR anti American OR Anti‐American rhetoric OR Global narrative OR Ideology, Ideological influence OR ideological legitimization OR Ideological message OR Ideological support OR Indoctrination OR Islamist extremist narrative OR Islamist ideology OR Jihadi ideologues OR Justifications for violence OR Legitimacy of terrorism OR Local narrative OR Master Narrative OR Media communication OR Message OR Message manipulation OR Meta‐narrative OR Misinformation OR Misinformation online OR Narrative OR narrative criminology OR Narrative transformation OR Observational Argument OR Online extremism OR opinion change OR Persuasion OR Persuasive communication OR Personal narrative OR Persuasive strategies OR Political idea OR Political strategy OR Propaganda campaign OR radicalism OR radical ideology OR radical narrative OR radical perspective OR radical perspectives OR radical theorist OR radical worldview OR recruitment narrative OR Religio‐ideological OR Religious justification OR Rhetoric OR Rhetorical tactics OR Rhetorical terrorism OR Rhetorical vision OR Social‐influence OR Statements OR Terror OR recruitment strategies)

**Table 2:** Electronic searches of bibliographic databases (stage 1)

**Database**	**Area**
**Web of Science**	*Science*
**PsycInfo**	*Psychology*
**Scopus**	*Science*
**Zetoc**	*Reports*
**Worldwide Political Science Abstracts**	Political Science
**Columbia International Affairs Online**	Politics
**Applied Social Sciences Index & Abstracts (ASSIA)**	Social Science
**EThOS**	Unpublished (Doctoral Theses)
**NCJRS Abstracts Database**	Criminal
**Directory of Open Access Journals (DOAJ)**	Science
**HedayahSecurity and Society)**	Security
**Canadian Network for Research on Terrorism**	Security

**Table 3:** Hand searches of several research and professional agencies’ outputs and publications (stage 2)

**Agency**	**Location**
**Institute for Strategic Dialogue (ISD)**	*London, United Kingdom*
**International Centre for Counter‐Terrorism (ICCT)**	*The Hague, Netherlands*
**European Consortium for Political Research (ECPR)**	*Colchester, United Kingdom*
**European Expert Network on Terrorism Issues (EENet)**	*German Federal Criminal Police Office*
**International Centre for the Study of Radicalisation (ICSR)**	*London, United Kingdom*

**Table 4:** Conceptual papers and books on the topic of counter‐narratives in counter‐terrorism (stage 3)

**Reference**
Briggs, R., &Reves, S. (2013). Review of programs to counter narratives of violent extremism: What works and what are the implications for government? : *Institute for Strategic Dialogue*.
Goodall Jr, H. (2010). *Counter‐narrative: How progressive academics can challenge extremists and promote social justice*. New York: Routledge.
Ferguson, K. (2016). Countering violent extremism through media and communication strategies: *The Partnership for Conflict, Crime and Security Research*.
Halverson, J. R., Corman, S.R., & Goodall Jr, H. L. (2011). *Master Narratives of Islamist Extremism*: Palgrave Macmillan US.
Tinnes, J. (2016). Bibliography: Terrorism and the Media (including the Internet) (Part 3).
Tinnes, J. (2014). Bibliography: Terrorism and the media (including the internet) (part 2). *Perspectives on Terrorism, 8*(6).
Tinnes, J. (2014). Bibliography: Terrorism and the Media (including the Internet) (Part 2). *Perspectives on Terrorism, 8*(6).
Tinnes, J. (2013). Literature on Terrorism and the Media (including the Internet): an Extensive Bibliography. *Perspectives on Terrorism, 7*(1).

**Table 5:** List of experts (stage 4)

**Name**	**Affiliation**
Neil Aggarwal	New York State Psychiatric Institute
JM Berger	International Centre for Counter Terrorism
Kurt Braddock	Pennsylvania State University
Marco de Swart	International Centre for Counter Terrorism (ICCT)
Paul Gill	University College London
John Horgan	Georgia State University
Niall O Dochartaigh	National University of Ireland, Galway
Alex Schmid	International Centre for Counter Terrorism (ICCT)
Anne Speckhard	International Center for the Study of Violent Extremism (ICSVE)
Judith Tinnes	Perspectives on Terrorism

## Appendix 2: Coding categories

*Coding lists*

a) Exclusion criteria for title and abstract screening
1) Exclude Duplicate
  a) Publications which match another title in exactly (i.e. title, author and year) should be excluded.
2) Exclude Language
  a) Publications not published in English should be excluded.
3) Exclude Publication Type
  a) Publications that do not report first‐hand empirical findings (e.g. government reports, newspaper articles and minutes of meetings) should be excluded.
4) Exclude non‐Intervention
  a) Publications which are not exposing participants to an intervention should be excluded.
  b) Publications which are not adopting an experimental design (e.g. RCT or factorial design) should be excluded.
5) Exclude Narrative vs. Counter‐Narrative
  a) Publications which do not challenge an existing narrative should be excluded.
6) Exclude Unrelated to Violent Radicalisation
  a) Publications which do not measure outcomes related to violent radicalisation or target risk factors for violent radicalisation should be excluded.
b) ***Inclusion criteria for title and abstract screening***
  1) Counter‐Narrative intervention
    a) Publications exposing participants to an intervention which can be operationally defined as a counter‐narrative should be included.
      i. The counter‐narrative intervention may be therapeutic (delivered following exposure to an existing narrative).
      ii. The counter‐narrative intervention may be preventative (delivered before exposure to a narrative ‘to‐be‐countered’)
  2) Evidence of existing narrative
    a) Publications which show evidence of an existing narrative or a narrative ‘to‐be‐countered’ should be included.
      i. The narrative may be determined by baseline/pre‐test scores.
      ii. The narrative may be experimentally introduced.
      iii. The narrative among the population may be empirically evident in a different study.
      iv. The narrative may be evident in the sample due considerable evidence to be weighed by the initial and second coder.
3) Outcomes
  a) Publications which measure outcomes related to violent radicalisation should be included.
    i. Outcomes may be primary outcomes related to violent radicalisation (e.g. engagement in violent extremism or providing support to violent extremist groups).
    ii. Outcomes may be secondary outcomes related to violent radicalisation (e.g. adversarial stereotypes, outgroup feelings or attitudes towards violence).
      ➢ Secondary outcomes, in particular, must have justification for inclusion as outlined in the outcomes justifications table (Table X).
4) Design
  a) Publications adopting an experimental design where at least one of the independent variables involves comparing a counter‐narrative to a control or comparison exposure should be included. These may include:
    i. Randomised Control Trials (RCT) whereby participants are randomly assigned to experimental or control conditions (e.g. two‐group between‐subjects design).
    ii. Factorial designs, with more than one independent variable (e.g. pre‐post as a within‐subjects variable, and exposure (e.g. present/absent) as a between‐subjects variable.
    iii. Single group pre‐ and post‐test studies which collect longitudinal data at baseline and end‐line

**Table:**

**a) Descriptive coding scheme**		
**Study and year**		Allhabash and Wise (2012)
**Participants**	*N* *Gender* *Ethnicity/nationality*	Pro‐Israeli participants (*N* = 68; 74% female; mean age = 20) recruited from an introductory undergraduate advertising course at an American university.
**Narrative being challenged**		Anti‐Palestinian narrative.
**Counter‐narrative**		Role play from the Palestinian perspective to initiate self‐persuasion
**Study design**		2 x 2 Factorial design
	*Theory*	The study exposed participants to a video game which was designed according to procedural rhetoric (Bogost, 2006; 2007) whereby message autonomy (the players capacity to exhibit agency over decisions in the game), integration (the embeddedness of the object of persuasion) and goal overlap (the level of overlap between the learning goal and the tactical goals of the game) are used to encourage self‐persuasion.
	*Intervention*	Participants were instructed to play the role of either the Palestinian president (experimental condition) or the Israeli prime minister (control condition) with the ultimate tactical goal of achieving peace.
	*Risk factors*	The counter‐narrative targeted negative attitudes towards Palestinians as a risk factor for ethnic extremism.
	*Logic*	The intervention followed the logic that through integrating persuasive elements into a video game, participants with previous negative attitudes towards Palestinians would report lower negative implicit attitudes, reducing their risk of engaging in ethnic extremism against Palestinians
**Outcomes (operationalised)**		Explicit attitudes towards the adversary (Palestinians).
**Outcomes (measured)**		Explicit attitudes were measured with a validated national attitudes scale which: measured 7 statements about each national group regarding (1) favourability; (2) sympathy; (3) belief about the national group's intention for peace; (4) intentionally targeting civilians from the other side; (5) being democratic; (6) being responsible for the violence and, (7) having the right to sole control over the city of Jerusalem.

**Table:**

b) Coding scheme representing effects of the intervention		
**Study and year**	Allhabash and Wise (2012)	
**Outcome**	Explicit attitudes towards an adversary measured using a validated national attitudes scale (α = .71). Higher scores = higher favourability towards the adversary (Palestinians).	
**Results**	*Control condition* (playing the role of the Israeli prime minister)	*Experimental condition* (playing the role of the Palestinian president)
	*N =* 33	*N =* 35
	*M = 3.77*	*M = 4.03*
	*SD = 0.47*	*SD = 0.49*
